# RNA Modification Profiles and m^6^A Regulatory Patterns in the Lungs of Sheep at Different Altitudes

**DOI:** 10.3390/ani16213393

**Published:** 2026-10-27

**Authors:** Yan Lei, Chao Yuan, Bowen Chen, Tingting Guo, Fan Wang, Wenhao Li, Zengkui Lu, Jianbin Liu

**Affiliations:** 1 Key Laboratory of Animal Genetics and Breeding on the Tibetan Plateau, Ministry of Agriculture and Rural Affairs, Lanzhou Institute of Husbandry and Pharmaceutical Sciences, Chinese Academy of Agricultural Sciences, Lanzhou 730050, China; 15293116355@163.com (Y.L.); yuanchao@caas.cn (C.Y.); chenbowen@caas.cn (B.C.); guotingting@caas.cn (T.G.); 2 Sheep Breeding Engineering Technology Research Center of Chinese Academy of Agricultural Sciences, Lanzhou 730050, China; 3 College of Life Science and Engineering, Northwest Minzu University, Lanzhou 730030, China; wangfaneasy@126.com; 4 Qilian Tibetan Sheep Industry Development Research Institute of Qinghai Province, Qilian 810400, China; qhdxlwh@163.com

**Keywords:** lung, hypoxia adaptation, epitranscriptomics, m^6^A, HIF signaling, Tibetan Plateau

## Abstract

The lung is a key organ for oxygen uptake and gas exchange and plays an important role in adaptation to hypoxic environments at high altitudes. RNA modifications represent an important layer of epitranscriptomic regulation and participate in cellular processes by affecting RNA stability, splicing, translation, and degradation. In this study, the right lung parenchyma from low-altitude Hu sheep (LL), mid-altitude Tibetan sheep (ML), and high-altitude Tibetan sheep (HL) were used to investigate altitude-associated RNA modification profiles and m^6^A regulatory patterns. A UPLC-MS/MS platform was employed to quantitatively detect 55 RNA modifications, while RT-qPCR and Western blot analyses were performed to evaluate the expression of HIF1α, HIF2α, and m^6^A-related regulatory factors. The results showed distinct differences in lung RNA modification profiles among the three groups. Compared with the LL and ML groups, the HL group exhibited reduced m^6^A and m^6^Am levels, whereas several non- m^6^A modifications, including t^6^A, m^1^G, m^2^G, m^7^G, m^3^C, and m^5^C, showed increasing trends. The expression levels of HIF1α and HIF2α were higher in the ML and HL groups than in the LL group. In addition, METTL3, METTL14, WTAP, and several YTH family reader proteins were downregulated in the HL group at both the protein and mRNA levels. These findings indicate that sheep lung RNA modification profiles and the m^6^A regulatory machinery differ among groups with different altitude-related adaptation backgrounds. Collectively, these findings reveal epitranscriptomic differences associated with altitude and breed in sheep lungs and provide a basis for further investigation of the role of m^6^A regulation in high-altitude adaptation.

## Simple Summary

High-altitude animals rely on lung adaptations to cope with hypoxia. We compared lung RNA modifications and m^6^A regulators among low-altitude Hu sheep and Tibetan sheep from different altitudes. High-altitude Tibetan sheep showed reduced m^6^A modification, increased levels of some other RNA modifications, and higher HIF1α and HIF2α expression. Several key m^6^A regulators were also decreased. These findings reveal altitude- and breed-associated differences in RNA modification profiles and identify reduced m^6^A abundance as a candidate molecular feature of high-altitude Tibetan sheep lungs.

## 1. Introduction

High-altitude environments are characterized by hypobaric hypoxia, low temperature, strong ultraviolet radiation, and marked climatic variability, all of which impose substantial physiological challenges on animals living in these regions [1]. Among these environmental pressures, hypoxia is considered a major factor driving physiological and molecular adaptation. As the primary organ responsible for oxygen uptake and gas exchange, the lung is directly involved in sensing and responding to reduced oxygen availability. Long-term adaptation to high-altitude environments is therefore accompanied by coordinated changes in pulmonary structure, oxygen transport, vascular regulation, and energy metabolism [2,3]. Tibetan sheep are indigenous livestock that have long inhabited the Qinghai–Tibet Plateau and surrounding high-altitude regions and have developed physiological and genetic characteristics associated with adaptation to harsh plateau environments. Previous studies based on genomics, transcriptomics, proteomics, and physiological analyses have identified a series of adaptive mechanisms involving oxygen sensing, angiogenesis, erythropoiesis, energy metabolism, and tissue remodeling [4,5,6,7,8]. Hypoxia-inducible factors (HIFs), especially HIF1α and HIF2α, play central roles in cellular responses to reduced oxygen availability by regulating genes involved in oxygen transport, glycolytic metabolism, vascular development, and cellular homeostasis [9,10,11]. Nevertheless, most studies of high-altitude adaptation in Tibetan sheep have focused on genomic variation and transcriptional or protein-level regulation. Whether post-transcriptional RNA modifications participate in pulmonary adaptation to different altitude-related environments remains much less understood.

RNA modifications are an important component of post-transcriptional regulation. To date, a wide range of chemical RNA modifications have been identified [12]. These modifications are widely distributed in mRNA, tRNA, rRNA, and non-coding RNAs, and can affect RNA structure, stability, splicing, nuclear export, translation, and degradation [13,14,15]. LC-MS/MS or UPLC-MS/MS enables quantitative detection of multiple RNA modifications at the nucleoside level and is commonly used to assess global RNA modification levels in epitranscriptomic studies [16,17]. Among these modifications, N^6^-methyladenosine (m^6^A) is the most extensively investigated internal modification of eukaryotic mRNA. The m^6^A modification is dynamically regulated by three major groups of proteins. The writer complex, including METTL3, METTL14, and WTAP, is responsible for m^6^A deposition; the erasers FTO and ALKBH5 mediate m^6^A demethylation; and reader proteins, particularly members of the YTH family such as YTHDF1-3 and YTHDC1-2, recognize m^6^A-modified transcripts and mediate their downstream effects [18,19,20]. Increasing evidence indicates that m^6^A modification interacts closely with cellular hypoxic responses. Hypoxia can alter the expression or activity of m^6^A regulatory factors, while m^6^A-dependent regulation can in turn influence the stability and translation of transcripts involved in HIF signaling, metabolism, angiogenesis, and cellular stress adaptation [21,22,23]. These observations suggest that RNA modification may provide an additional regulatory layer linking environmental oxygen availability with gene expression. However, current knowledge has largely been derived from cellular or disease-related models, whereas information on RNA modification profiles in the lungs of high-altitude livestock remains limited.

Previous studies on Tibetan sheep have provided important insights into high-altitude adaptation through physiological, genomic, transcriptomic, and proteomic analyses. However, these studies have mainly focused on gene sequence variation, transcriptional responses, protein expression, and pulmonary physiological changes. The contribution of post-transcriptional RNA modifications, particularly the global RNA modification landscape and the m^6^A regulatory system, remains poorly characterized in sheep lung tissue. In addition, most previous studies have not simultaneously evaluated multiple RNA modification types together with key m^6^A writers, erasers, and readers. Therefore, the present study provides an exploratory assessment of pulmonary RNA modification profiles and m^6^A-related regulatory patterns across sheep groups with different altitude-related backgrounds. In the present study, lung tissues from low-altitude Hu sheep, mid-altitude Tibetan sheep, and high-altitude Tibetan sheep were examined. UPLC-MS/MS was used to quantify 55 RNA modifications, while RT-qPCR and Western blotting were employed to assess the expression patterns of HIF1α, HIF2α, and m^6^A regulatory factors. This study aimed to characterize differences in pulmonary RNA modification profiles and the m^6^A regulatory network among sheep groups with different altitude-related backgrounds, thereby providing new evidence for understanding the epitranscriptomic regulatory mechanisms underlying high-altitude adaptation in Tibetan sheep lung tissue.

## 2. Materials and Methods

### 2.1. Animal and Sample Collection

All animal procedures in this study were conducted in accordance with the relevant animal ethics regulations of the People’s Republic of China and were approved by the Animal Management and Ethics Committee of the Animal Ethics Committee of the Lanzhou Institute of Husbandry and pharmaceutical science of Chinese Academy of Agricultural Sciences (approval No. 2026-11). Based on the altitude of the sampling sites and the breed background of the animals, the experimental sheep were divided into three groups, with three animals in each group and nine animals in total. The low-altitude group (LL) consisted of three Hu sheep from Minqin County, Gansu Province, China, at approximately 1400 m above sea level (approximately 38.62° N, 103.09° E). The mid-altitude group (ML) consisted of three Tibetan sheep from Xiahe County, Gansu Province, China, at approximately 3238 m above sea level (approximately 35.20° N, 102.52° E). The high-altitude group (HL) consisted of three Tibetan sheep from Amdo (Anduo) County, Xizang Autonomous Region, China, at approximately 4800 m above sea level (approximately 32.26° N, 91.68° E). Three yearling rams of similar physical condition were selected for each group. Due to the difficulty of obtaining high-altitude lung tissue samples, three biological replicates were included in each group. All animals were maintained under local free-grazing conditions. Before slaughter, the selected sheep were fasted for at least 12 h, with water withheld for 3 h. Slaughter and related procedures were performed in accordance with the National Standard of the People’s Republic of China (GB/T 43562-2023) [24]. The right lung parenchyma were immediately frozen in liquid nitrogen and then transferred to −80 °C for storage until subsequent RNA extraction, protein extraction, and RNA modification analysis.

### 2.2. RNA Modification Detection and Analysis

Ultra-performance liquid chromatography-tandem mass spectrometry (UPLC-MS/MS) was used to quantitatively analyze 55 modified nucleosides in RNA extracted from lung tissues. Briefly, total RNA was first extracted from lung tissues, and its concentration and purity were assessed. For each sample, 1 μg of RNA was incubated with S1 nuclease (Takara, Japan; 180 U/μL), phosphodiesterase I (Sigma-Aldrich, St. Louis, MO, USA; 0.002 U/μL), and alkaline phosphatase (Takara, Shiga, Japan; 30 U/μL) at 37 °C to achieve complete enzymatic digestion of RNA into mononucleosides. The digested products were extracted with chloroform, and the upper aqueous phase was collected and transferred to injection vials for UPLC-MS/MS analysis. Chromatographic separation was performed using a Waters ACQUITY UPLC HSS T3 C18 column (Waters, Milford, MA, USA). Mobile phase A consisted of ultrapure water containing 2 mM ammonium bicarbonate, and mobile phase B consisted of methanol containing 2 mM ammonium bicarbonate. Gradient elution was performed according to the established program, with a flow rate of 0.30 mL/min, a column temperature of 40 °C, and an injection volume of 10 μL. Mass spectrometric detection was conducted using an electrospray ionization source. The ion source temperature was set at 550 °C, the voltage in positive ion mode was 5500 V, and the curtain gas pressure was 35 psi. Target ion pairs were detected using a Q-Trap 6500+ mass spectrometer (SCIEX, Framingham, MA, USA) in multiple reaction monitoring mode. Standard curves were established using standard solutions with different molar amounts, with the molar amount of each nucleoside plotted on the *x*-axis and the corresponding peak area plotted on the *y*-axis. Qualitative identification was performed based on a MWDB database established using authentic standards, and quantitative analysis was carried out using multiple reaction monitoring on a triple quadrupole mass spectrometer. Mixed quality control samples were used to evaluate the stability and reproducibility of the detection method.

### 2.3. Protein Extraction and Western Blot Analysis

An appropriate amount of lung tissue was homogenized in RIPA lysis buffer containing 1% PMSF (Beyotime Biotechnology, Shanghai, China) and lysed on ice for 30 min. The lysates were then centrifuged, and the supernatants were collected. Protein concentrations were determined using the BCA assay. Briefly, 1 μL of each protein sample was diluted with 19 μL of PBS and added to a 96-well plate. BCA reagents A and B were mixed at a ratio of 50:1 (*v*/*v*), and 200 μL of the resulting working reagent was added to each well. After incubation at 37 °C for 30 min, absorbance was measured at 562 nm using a microplate reader. Protein concentrations were calculated based on the standard curve. Subsequently, SDS-PAGE Sample Loading Buffer (Beyotime Biotechnology, Shanghai, China) was added to the protein samples, followed by denaturation in a boiling water bath for 10 min. Equal amounts of protein were separated by SDS-PAGE and transferred onto PVDF membranes. The membranes were blocked with 5% skim milk and then washed with TBST. Primary antibodies were added, and the membranes were incubated overnight at 4 °C. After washing with TBST on the following day, the membranes were incubated with the corresponding secondary antibodies at room temperature. Protein bands were visualized using an ECL chemiluminescence reagent, and images were captured using a gel imaging system. The integrated optical density of each target protein band was quantified using ImageJ software (version 1.54d, National Institutes of Health, Bethesda, MD, USA). ACTB was used as the internal reference protein, and the relative expression levels of target proteins were calculated accordingly. β-Actin (ACTB) was used as the internal reference protein (Table 1).

### 2.4. RNA Extraction and Quantitative Real-Time PCR

Total RNA was extracted from lung tissues using TRIzol reagent (Takara, Japan), and RNA purity and concentration were then assessed. RNA concentration and purity were evaluated using a NanoDrop spectrophotometer by measuring the absorbance at 260 nm and the A260/A280 ratio. The extracted RNA was reverse-transcribed into cDNA according to the manufacturer’s instructions of the reverse transcription kit (TransGen Biotechnology, Beijing, China). Quantitative real-time PCR was performed using cDNA as the template. Each 20 μL reaction mixture contained TransStart^®^ Top Green qPCR SuperMix (TransGen Biotechnology, Beijing, China), forward and reverse primers, cDNA template, and ddH_2_O. Amplification was carried out on a Roche LightCycler^®^ 96 real-time PCR system under the following conditions: 94 °C for 30 s, followed by 40 cycles of 94 °C for 5 s, 60 °C for 15 s, and 72 °C for 10 s. ACTB was used as the internal reference gene, and the relative expression levels of target genes were calculated using the 2^−ΔΔCt^ method. Detailed information on the primers used in this study is listed in Table 2.

### 2.5. Statistical Analysis

All statistical analyses were performed using GraphPad Prism 8.0 software. Because each group contained only three biological replicates, the normality assumption could not be reliably assessed. Therefore, data were summarized descriptively, and group differences were analyzed using the non-parametric Kruskal–Wallis test. When appropriate, Dunn’s multiple comparisons test was used for post hoc pairwise comparisons. The value of *p* < 0.05 was considered statistically significant.

## 3. Results

### 3.1. Detection Standard Curves and Stability of Detection Conditions

To evaluate the stability of the UPLC-MS/MS detection system, equal amounts of extracts from the three groups were pooled to prepare quality control samples, which were repeatedly analyzed under the same instrumental conditions. The results showed that the total ion chromatograms of the pooled quality control samples exhibited high overlap in both positive and negative ion modes. The retention times and peak intensities of the major chromatographic peaks were highly consistent, indicating good stability of the sample preparation procedure and instrumental acquisition conditions. The correlation coefficients of the standard curves for all 55 RNA modifications were greater than 0.99, demonstrating that this method met the requirements for quantitative analysis of modified nucleosides in lung RNA (Table S1). These results indicate that the RNA modification data obtained in this study had good reproducibility and reliability and were suitable for subsequent comparative analysis among groups.

### 3.2. Distinct Lung RNA Modification Profiles Among Sheep Groups with Different Altitude-Related Backgrounds

**Figure 1:**
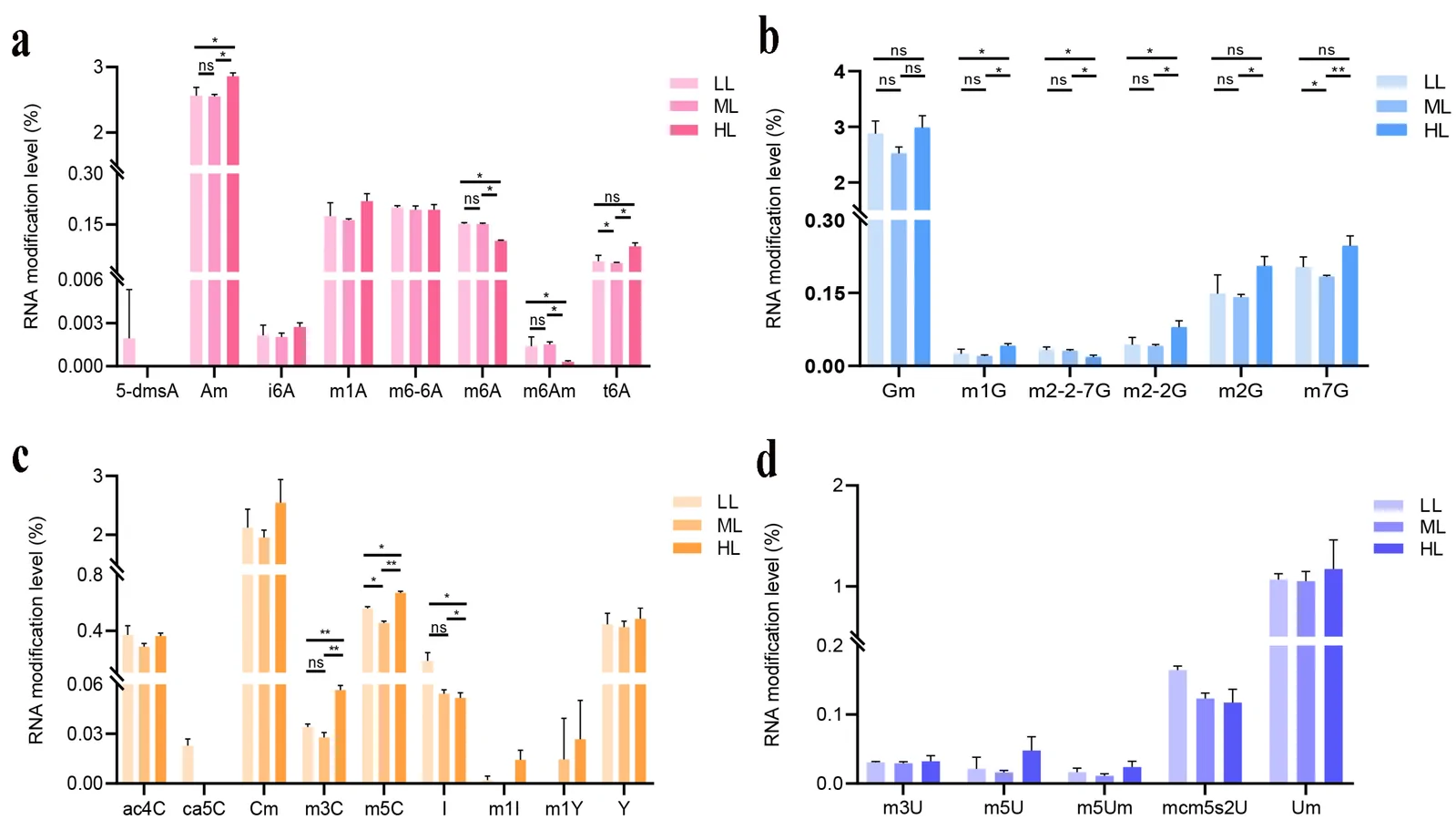
UPLC-MS/MS quantification of RNA modification profiles in sheep lungs from different groups. (**a**–**d**) Relative levels of different RNA modifications in the lungs of low-altitude Hu sheep (LL), middle-altitude Tibetan sheep (ML), and high-altitude Tibetan sheep (HL). (**a**) Adenosine-related modifications; (**b**) guanosine-related modifications; (**c**) cytidine-related modifications; (**d**) uridine-related modifications. The RNA modification level (relative quantities of modified ribonucleosides) = modified ribonucleosides/canonical ribonucleosides. Data are presented as mean ± SEM (*n* = 3 biological replicates per group). Statistical significance between groups is indicated as *; *p* < 0.05, **; *p* < 0.01, ns: no significant difference.

Based on the UPLC-MS/MS platform, 55 types of RNA modifications were quantitatively analyzed in lung RNA samples from the LL, ML, and HL groups. The results showed clear differences in lung RNA modification profiles among the three groups. The direction and magnitude of changes varied among different modification types under different altitude-related ecological adaptation backgrounds, indicating strong modification-specific patterns. At the global modification level, Am, Gm, Cm, Um, and other related modifications were highly abundant in lung RNA, suggesting that ribose methylation constitutes an important component of the RNA modification profile in sheep lung tissue. Among adenosine-related modifications, m^6^A and m^6^Am showed marked differences among groups. Compared with the LL and ML groups, the HL group exhibited a significant decrease in lung m^6^A levels, while m^6^Am also showed a downward trend, suggesting that m^6^A-related modifications may be suppressed in the lungs of high-altitude Tibetan sheep. In contrast, t6A was increased in the HL group, indicating that different adenosine-derived modifications did not change synchronously but instead exhibited divergent regulatory patterns (Figure 1a). Among guanosine-related modifications, Gm was the dominant high-abundance modification, with no significant difference among the three groups. m^1^G, m^2^G, and m^7^G generally showed increasing trends in the HL group, with m^7^G exhibiting a more pronounced increase. In contrast, m^2-2-7^G showed a change pattern different from those of m^1^G and m^7^G (Figure 1b). Among cytidine-related modifications, ac4C and Cm showed relatively small overall changes, whereas m^3^C and m^5^C were more prominently increased in the HL group (Figure 1c). Among uridine-related modifications, m^3^U, m^5^U, and m^5^Um were generally present at low levels, with limited differences among groups. mcm^5^s^2^U showed a decreasing trend in the ML and HL groups, whereas Um, as a high-abundance modification, remained relatively stable among the three groups (Figure 1d). Overall, the lung RNA modification profiles of the LL, ML, and HL groups did not show a simple overall increase or decrease. Instead, they exhibited differential remodeling across specific modification types. The HL group was mainly characterized by decreased m^6^A and m^6^Am levels and increased levels of t^6^A, m^1^G, m^2^G, m^7^G, m^3^C, and m^5^C, suggesting that epitranscriptomic remodeling associated with RNA metabolism and translational regulation may occur in the lungs of high-altitude Tibetan sheep.

### 3.3. Increased HIF1α and HIF2α Expression in Tibetan Sheep Lungs

**Figure 2:**
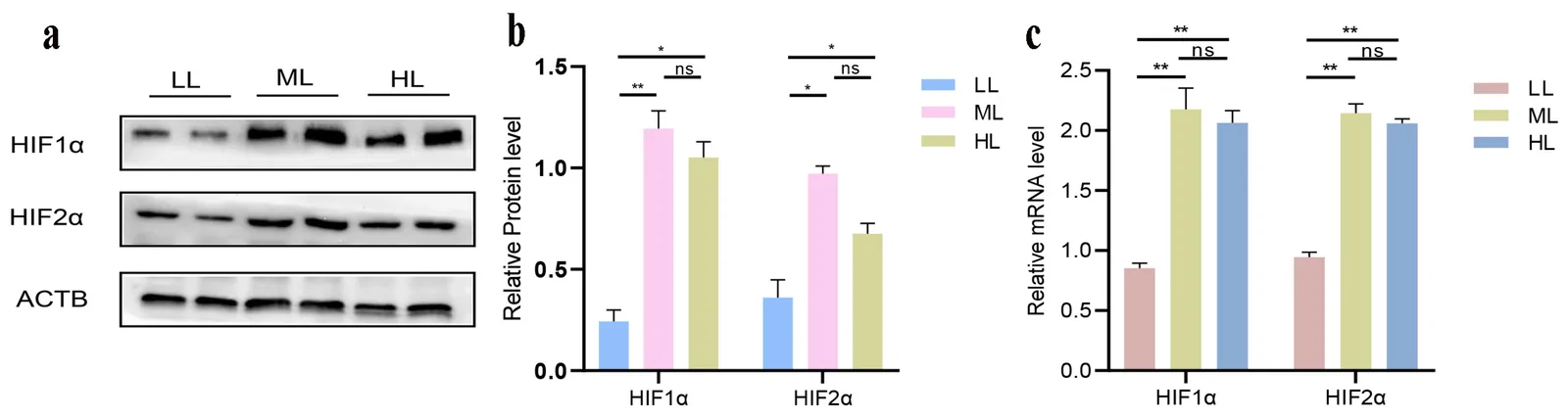
Protein and mRNA expression levels of HIF1α and HIF2α in sheep lungs from different groups. (**a**) Western blot analysis of HIF1α and HIF2α protein expression in the lungs of LL, ML, and HL groups, with ACTB used as the internal reference protein; (**b**) statistical analysis of relative protein expression levels of HIF1α and HIF2α; (**c**) RT-qPCR analysis of relative mRNA expression levels of *HIF1α* and *HIF2α*. Statistical significance between groups is indicated as *; *p* < 0.05, **; *p* < 0.01, ns: no significant difference.

To evaluate the hypoxic response status of sheep lung tissues among groups with different altitude-related backgrounds, the protein and mRNA expression levels of HIF1α and HIF2α were examined. Western blot analysis showed that, compared with the LL group, both the ML and HL groups exhibited increased protein expression levels of HIF1α and HIF2α. Among them, the ML group showed relatively higher HIF1α and HIF2α protein expression, whereas the HL group showed lower levels than the ML group but still higher levels than the LL group (Figure 2a,b). RT-qPCR results showed that the mRNA expression levels of *HIF1α* and *HIF2α* were significantly higher in both the ML and HL groups than in the LL group, while no obvious difference was observed between the ML and HL groups (Figure 2c). These results indicate that hypoxia-responsive signaling was enhanced in sheep lung tissues from the mid- and high-altitude-related groups. The higher expression of HIF1α and HIF2α in the ML and HL groups compared with the LL group suggests that Tibetan sheep lungs may be in a more active state of oxygen sensing and hypoxic response.

### 3.4. Reduced m^6^A Writer Expression in High Altitude Tibetan Sheep

**Figure 3:**
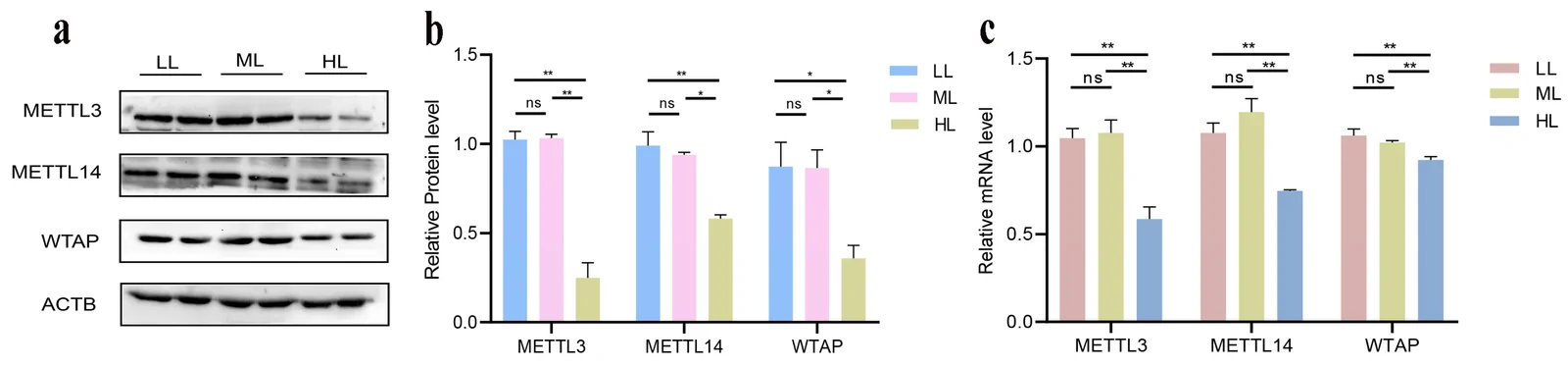
Protein and mRNA expression levels of m^6^A methyltransferase-related factors in sheep lungs from different groups. (**a**) Western blot analysis of METTL3, METTL14, and WTAP protein expression in the lungs of LL, ML, and HL groups, with ACTB used as the internal reference protein; (**b**) statistical analysis of relative protein expression levels of METTL3, METTL14, and WTAP; (**c**) RT-qPCR analysis of relative mRNA expression levels of *METTL3*, *METTL14*, and *WTAP*. Statistical significance between groups is indicated as *; *p* < 0.05, **; *p* < 0.01, ns: no significant difference.

To further explain the potential causes of changes in m^6^A modification levels, the expression levels of METTL3, METTL14, and WTAP, the core components of the m^6^A methyltransferase complex, were examined. Western blot analysis showed that the protein expression levels of METTL3, METTL14, and WTAP in the HL group were markedly lower than those in the LL and ML groups, with more pronounced decreases observed for METTL3 and WTAP (Figure 3a,b). The overall differences between the ML and LL groups were relatively small, suggesting that the m^6^A writer system was not obviously suppressed in the lungs of mid-altitude Tibetan sheep but was markedly reduced in those of high-altitude Tibetan sheep. The RT-qPCR results were generally consistent with the protein expression results. The mRNA expression levels of *METTL3*, METTL14, and *WTAP* in the HL group were lower than those in the LL and ML groups, with more evident decreases in *METTL3* and *METTL14* (Figure 3c). METTL3 and METTL14 constitute the core catalytic structure of the m^6^A methyltransferase complex, while WTAP is involved in the localization and stabilization of the complex. Therefore, the coordinated downregulation of METTL3, METTL14, and WTAP in the HL group may be associated with the reduced m^6^A modification level observed in high-altitude Tibetan sheep lungs.

### 3.5. Group-Specific Changes in the Expression of the m^6^A Demethylases FTO and ALKBH5

**Figure 4:**
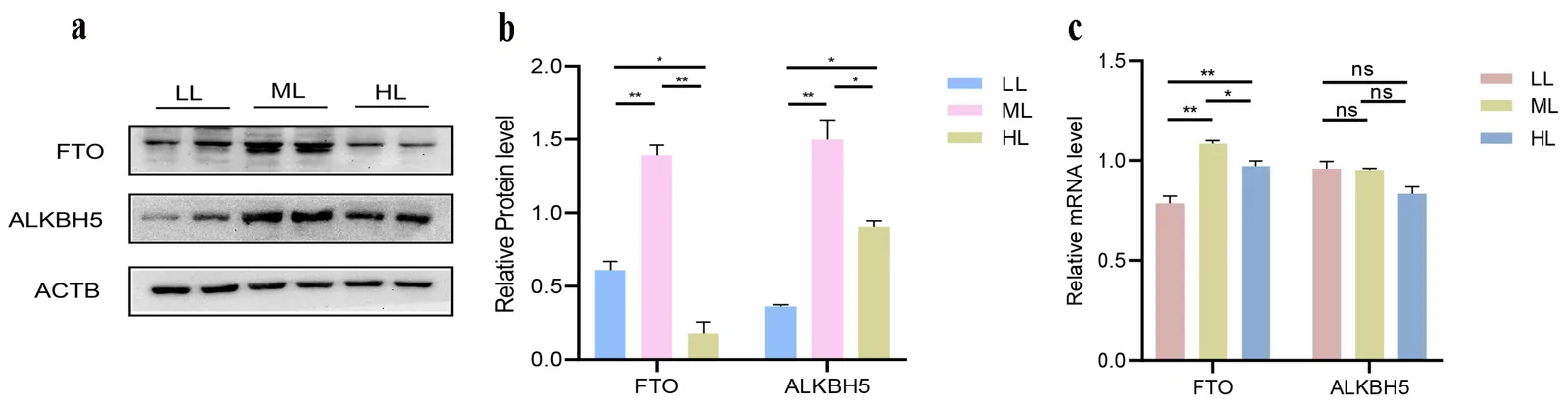
Protein and mRNA expression levels of m^6^A demethylase-related factors in sheep lungs from different groups. (**a**) Western blot analysis of FTO and ALKBH5 protein expression in the lungs of LL, ML, and HL groups, with ACTB used as the internal reference protein; (**b**) statistical analysis of relative protein expression levels of FTO and ALKBH5; (**c**) RT-qPCR analysis of relative mRNA expression levels of *FTO* and *ALKBH5*. Statistical significance between groups is indicated as *; *p* < 0.05, **; *p* < 0.01, ns: no significant difference.

To analyze changes in the removal process of m^6^A modification, the protein and mRNA expression levels of FTO and ALKBH5 were examined. Western blot analysis showed that FTO and ALKBH5 protein levels were significantly increased in the ML group but markedly decreased in the HL group. Specifically, FTO protein expression in the HL group was lower than that in both the LL and ML groups, whereas ALKBH5 protein expression in the HL group was higher than that in the LL group but markedly lower than that in the ML group (Figure 4a,b). The RT-qPCR results showed that FTO mRNA expression was highest in the ML group, while its expression in the HL group was slightly lower than that in the ML group but remained higher than that in the LL group. In contrast, ALKBH5mRNA expression did not differ significantly among the three groups (Figure 4c). These results indicate that the expression patterns of FTO and ALKBH5 were not fully consistent between the protein and mRNA levels, suggesting that they may be influenced by post-transcriptional regulation, protein stability, or translational efficiency.

### 3.6. Differential Expression of m^6^A Reader Proteins Among Sheep Groups with Different Altitude-Related Backgrounds

**Figure 5:**
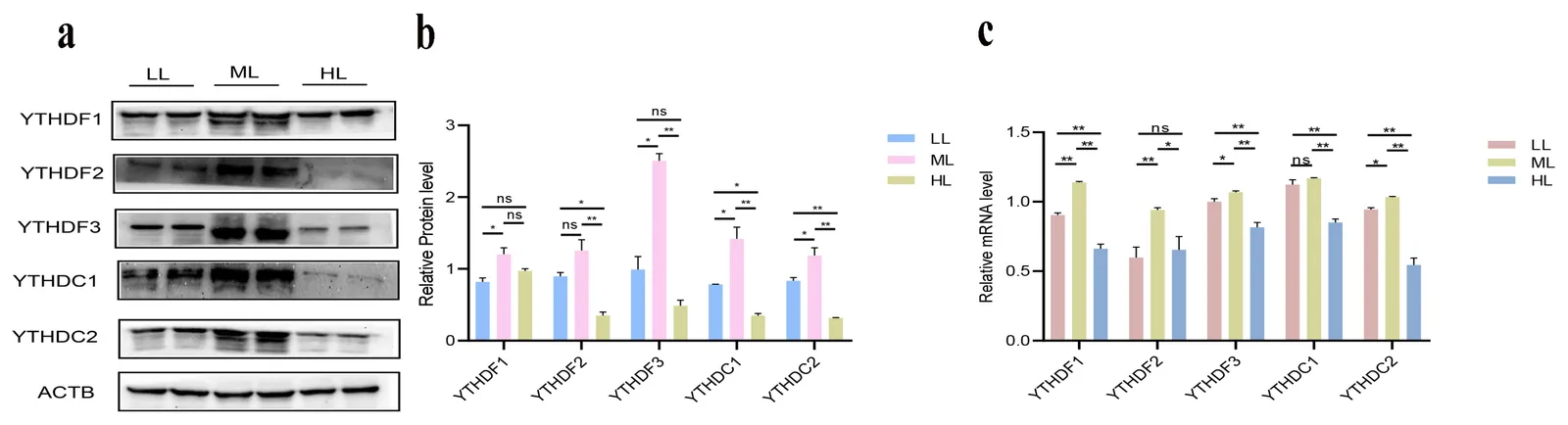
Protein and mRNA expression levels of m^6^A reader proteins in sheep lungs from different groups. (**a**) Western blot analysis of YTHDF1, YTHDF2, YTHDF3, YTHDC1, and YTHDC2 protein expression in the lungs of LL, ML, and HL groups, with ACTB used as the internal reference protein; (**b**) statistical analysis of relative protein expression levels of YTHDF1, YTHDF2, YTHDF3, YTHDC1, and YTHDC2; (**c**) RT-qPCR analysis of relative mRNA expression levels of *YTHDF1*, *YTHDF2*, *YTHDF3*, *YTHDC1*, and *YTHDC2*. Statistical significance between groups is indicated as *; *p* < 0.05, **; *p* < 0.01, ns: no significant difference.

To further investigate the reading process of m^6^A modification signals, the protein and mRNA expression levels of YTHDF1, YTHDF2, YTHDF3, YTHDC1, and YTHDC2 were examined. Western blot analysis showed that several YTH family proteins were upregulated in the ML group, especially YTHDF3, YTHDC1, and YTHDC2. In contrast, multiple YTH proteins were downregulated in the HL group, and the expression levels of YTHDF2, YTHDF3, YTHDC1, and YTHDC2 were lower than those in the ML group. YTHDF1 increased in the ML group, whereas the difference between the HL and LL groups was relatively small (Figure 5a,b). At the mRNA level, most YTH family genes showed higher expression in the ML group, whereas most of them were downregulated in the HL group. Among them, the expression levels of *YTHDF1*, *YTHDF3*, *YTHDC1*, and *YTHDC2* in the HL group were lower than those in the ML group (Figure 5c). These results indicate that the m^6^A reader system in sheep lung tissues differed markedly among groups with different altitude-related backgrounds. The ML group may exhibit a more active m^6^A reading state, whereas the HL group showed an overall downregulation of m^6^A reader proteins.

## 4. Discussion

In this study, UPLC-MS/MS was used to systematically quantify 55 types of RNA modifications in lung RNA samples from the LL, ML, and HL groups, and clear differences in RNA modification profiles were observed among the three groups. As an important layer of epitranscriptomic regulation, RNA modifications can participate in the regulation of gene expression by affecting RNA stability, structure, splicing, translation, and degradation [14,15]. In the present study, Am, Gm, Cm, Um, and related modifications were highly abundant in lung RNA, suggesting that ribose methylation may represent a major component of the RNA modification profile in sheep lung tissue. The levels of m^6^A and m^6^Am were decreased in the lungs of the HL group, whereas the levels of m^1^G, m^2^G, m^7^G, m^3^C, m^5^C, and t^6^A were increased, indicating that RNA modification changes in the lungs of high-altitude Tibetan sheep were not characterized by a global increase or decrease, but rather by modification type-specific remodeling. m^6^Am is mainly located adjacent to the mRNA 5′ cap at transcription start sites. It is catalyzed by PCIF1/CAPAM and can be demethylated by FTO, thereby participating in mRNA stability, decamping-mediated degradation, and post-transcription regulation [25,26,27,28,29,30]. In this study, the decreased level of m^6^Am in the HL group suggests that high-altitude hypoxia may affect the homeostasis of mRNA cap-associated modifications, thereby altering the stability and expression duration of specific transcripts. In contrast, m^1^G, m^2^G, m^7^G, m^3^C, m^5^C, and t^6^A were elevated in the HL group. Several of these modified nucleosides, particularly m^1^G, m^2^G, m^7^G, m^3^C, m^5^C, and t^6^A, are commonly associated with tRNAs and have been implicated in tRNA structural stability, decoding, translation fidelity, and cellular stress responses [31,32,33,34,35]. The remodeling of RNA modification profiles observed in this study suggests that the lung tissues of high-altitude Tibetan sheep may not respond to hypoxic stress simply by enhancing or suppressing a single class of RNA modification. Instead, they may achieve layered regulation of mRNA stability and tRNA-mediated translation through coordinated changes among different types of RNA modifications.

HIF1α and HIF2α are core regulators of the hypoxic response and mediate adaptive responses to changes in oxygen partial pressure [11]. Under hypoxic conditions, degradation of HIFα subunits is inhibited, leading to their intracellular accumulation. HIFα then forms transcriptional complexes with HIFβ, thereby regulating the expression of genes involved in angiogenesis, erythropoiesis, glycolytic metabolism, mitochondrial function, and oxygen transport [9,10]. In the present study, the protein and mRNA expression levels of HIF1α and HIF2α in the lungs of the ML and HL groups were higher than those in the LL group, indicating enhanced hypoxia-responsive signaling in lung tissues from mid- and high-altitude-related groups. This finding is consistent with the long-term exposure of Tibetan sheep to high-altitude hypoxic environments and with the physiological role of the lung as the central organ for oxygen uptake and gas exchange. Previous studies on Tibetan sheep have shown that adaptation to high-altitude hypoxia involves coordinated remodeling of multiple organs, with the lung playing a key role in this process [4,5]. Therefore, the increased expression of HIF1α and HIF2α observed in this study may serve as molecular evidence for the activation of hypoxic responses in lung tissue. However, the protein levels of HIF1α and HIF2α in the HL group were not higher than those in the ML group, suggesting that high-altitude adaptation is not simply a linearly enhanced process [3,36]. HIF regulation occurs at multiple levels. HIF mRNA abundance, protein stability, and downstream transcriptional activity may not change in parallel because HIFα proteins are strongly controlled by oxygen-dependent post-translational stabilization and degradation. Therefore, the observed expression patterns indicate group-specific differences in HIF regulation, but the physiological significance of these differences requires further functional and longitudinal investigation.

m^6^A modification is one of the most common internal modifications in mammalian mRNA, and its dynamic balance is jointly maintained by the writing, erasing, and reading systems [18,19,20]. In this study, m^6^A modification levels were reduced in the lungs of the HL group, accompanied by marked downregulation of METTL3, METTL14, and WTAP at both the protein and mRNA levels. These results suggest that the reduced m^6^A level in the lungs of high-altitude Tibetan sheep may be mainly associated with weakened methylation writing capacity. Compared with measuring m^6^A levels alone, simultaneous detection of writer protein expression provides more direct regulatory evidence for the decrease in m^6^A modification. Downregulation of *METTL3*, *METTL14* and *WTAP* may reduce m^6^Adeposition on certain mRNAs in lung tissue, thereby affecting their stability, translational efficiency, or degradation rate. This change may have adaptive significance. In high-altitude hypoxic environments, lung tissue needs to regulate many transcripts related to oxygen sensing, vascular regulation, energy metabolism, and tissue remodeling. Reduced m^6^A levels may alter the life cycle of these transcripts, allowing lung tissue to establish a new balance in RNA metabolism under long-term hypoxic conditions. Previous studies have shown that hypoxia can interact with the m^6^A pathway, and that m^6^A epitranscriptomic reprogramming can influence cellular adaptation to hypoxia and HIF-related regulation [21,22]. However, the present study mainly examined global m^6^A modification levels and the expression of m^6^A regulatory factors, and specific target transcripts have not yet been identified. Therefore, the hypoxia-responsive, energy metabolism-related, angiogenesis-related, or lung tissue remodeling-related genes affected by reduced m^6^A levels in the HL group remain to be further clarified through integrated analyses using MeRIP-seq, m^6^A-seq, and transcriptome sequencing.

The expression levels of several YTH family reader proteins, as well as the m^6^A demethylases FTO and ALKBH5, were increased in the ML group, whereas multiple YTH family proteins and demethylases decreased in the HL group. These patterns should not be interpreted as simple altitude-dependent increases or decreases. Instead, they may reflect group-specific regulation influenced by multiple factors, including breed background, environmental conditions, and post-transcriptional or protein-level regulation. Because the LL group differed in breed from the ML and HL groups, altitude-related effects cannot be separated completely from breed-associated effects. In the ML group, the increased expression of HIF1α and HIF2α, together with the upregulation of m^6^A reader proteins and demethylases, may suggest that the lungs of mid-altitude Tibetan sheep were in a relatively active state of dynamic RNA regulation. In contrast, the HL group showed decreased expression of m^6^A writer proteins, several reader proteins, and demethylases, which may indicate that the m^6^A regulatory system had shifted to a relatively low-level but stable state after long-term high-altitude adaptation. In addition, the mRNA and protein expression patterns of FTO and ALKBH5 were not fully consistent, suggesting that m^6^A demethylases may be influenced by post-transcriptional regulation, protein stability, or translational efficiency. Previous studies have shown that FTO and ALKBH5 play important roles in m^6^A demethylation, but their substrate preferences, subcellular localization, and biological functions are not identical [37,38]. Therefore, when interpreting the results for FTO and ALKBH5, changes in mRNA expression should not be simply equated with actual demethylase activity. Further validation is needed through enzyme activity assays and analysis of m^6^A modification levels on target genes.

The results of this study suggest that lung RNA modification profiles and the m^6^A regulatory network differ markedly among sheep groups with different altitude-related backgrounds. In particular, the HL group was characterized by decreased m^6^A levels, reduced expression of METTL3, METTL14 and WTAP, downregulation of several YTH family reader proteins, and increased expression of HIF1α and HIF2α. These changes may be associated with the adaptation of Tibetan sheep lung tissue to high-altitude hypoxic environments. However, it must be emphasized that altitude is not the only factor influencing the results of this study. The LL group consisted of low-altitude Hu sheep, whereas the ML and HL groups consisted of Tibetan sheep. Therefore, differences in breed genetic background, long-term domestication history, feeding environment, nutritional status, and regional ecological conditions may all have contributed to the observed intergroup differences. Accordingly, the molecular differences observed in this study should be interpreted as comprehensive differences associated with altitude-related ecological environments and breed adaptation backgrounds, rather than as direct causal effects of altitude alone. However, the protein levels of HIF1α and HIF2α in the HL group were not higher than those in the ML group.

## 5. Limitations

This study has several limitations. First, the sample size was relatively small, which may have limited the statistical power of the analysis. Second, the LL group consisted of Hu sheep, whereas the ML and HL groups consisted of Tibetan sheep. Therefore, the observed differences may have been influenced simultaneously by breed background, ecological conditions, and altitude-related environmental stress. Third, this study mainly focused on global RNA modification levels and expression patterns of m^6^A regulatory factors, whereas specific target transcripts and their functional consequences remain unclear. Future studies combining transcriptome sequencing, MeRIP-seq, and cellular hypoxia models are needed to further elucidate the molecular mechanisms underlying epitranscriptomic remodeling during high-altitude adaptation. Fourthly, because the UPLC-MS/MS analysis was performed on total RNA, the detected changes represent global nucleoside-level modification abundance and cannot be assigned to specific RNA classes or individual transcripts.

## 6. Conclusions

This study reveals that pulmonary RNA modification in sheep is characterized by coordinated, modification-specific remodeling rather than a uniform response across altitude-related adaptation backgrounds. The m^6^A regulatory system appears to be an important component of this remodeling and may participate in the long-term adjustment of pulmonary RNA metabolism under hypoxic conditions. This study is exploratory in nature, and these findings mainly provide complementary epitranscriptomic evidence for understanding altitude-associated molecular changes. Nevertheless, because altitude and breed background were not fully separated in the present design, the observed molecular patterns should be interpreted as altitude-associated rather than altitude-causal. Further validation in larger and genetically controlled populations will be required to determine their functional significance and potential breeding relevance.

## Figures and Tables

**Table 1:** The antibodies used in this study for Western blot.

Target Protein	Source	Item No	Western Blot Dilution Ratio
HIF1α	Proteintech	20960-1-AP	1:5000
HIF2α	Proteintech	26422-1-AP	1:2000
METTL3	Proteintech	15073-1-AP	1:5000
METTL14	Proteintech	26158-1-AP	1:4000
WTAP	Proteintech	60188-1-Ig	1:5000
FTO	Bioss	bs-7056R	1:1000
ALKBH5	Proteintech	16837-1-AP	1:5000
YTHDF1	Proteintech	17479-1-AP	1:5000
YTHDF2	Proteintech	24744-1-AP	1:5000
YTHDF3	Proteintech	25537-1-AP	1:5000
YTHDC1	Proteintech	14392-1-AP	1:1000
YTHDC2	Proteintech	27779-1-AP	1:5000
ACTB	Proteintech	20536-1-AP	1:5000
ACTB	Proteintech	20536-1-AP	1:5000

**Table 2:** Information on RT-qPCR primer sequences.

Genes	RefSeq Transcript Accession No	Forward Primer Sequence	Reverse Primer Sequence
*ACTB*	NM_009784.4	CTTCCAGCCTTCCTTCCTGG	GCCAGGGCAGTGATCTCTTT
*HIF1α*	XM_027971913.2	CTCACCCAACAGAGCAGGAA	TTCCTCAGGAGCAGTAGTTCTTT
*HIF2α*	XM_015094403.3	TTGGAGGGTTTCATTGCCGT	TCTTCCCAAAACCAGAGCCATT
*METTL3*	XM_04010362.6	AGATAGAGAGCCTTCTTAACCA	TCACTGGCTTTCATACACTCC
*METTL14*	XM_04009592.6	ATATTACAGAGAGACCGGCAT	CAATCCTTCCCCAGAACCACA
*WTAP*	XM_27972629.3	GCTTAAAAGCAGTCAGGATGAACT	GCGTCTCCTTCAGTTGTTGC
*FTO*	NM_01104931.1	TCCATCCTCGCCTCGATCAC	AATTCGAGACTGGCACCTGGC
*ALKBH5*	XM_04023544.5	GAAATCACCCACTGCATCCG	GGGTGCATCTAATCTCGTCTTCC
*YTHDF1*	XM_15099784.3	CGTGGACCCCCAGAGAACAA	GGCCATGGAGGGGTAACTGT
*YTHDF2*	XM_27965411.2	GCTACAAGCACACCACTTCC	CACGACCTTGACGTTCCTTT
*YTHDF3*	XM_60393720.1	CCTGTAATTCTACTCGTGGTGA	AAATATTCTGCTGCACGTTG
*YTHDC1*	XM_27971037.2	AAGCAGTGCCTCCAGAGAAC	CACTTCAAGCCCAATTCTCTCA
*YTHDC2*	XM_15096775.3	CATCTTTTCGTGCGGATGGT	GCTGCCTCTGGCTCTAGTTT

## Data Availability

The original contributions presented in this study are included in the article/Supplementary Material. Further inquiries can be directed to the corresponding authors.

## Supplementary Materials

The following supporting information can be downloaded at https://www.mdpi.com/article/10.3390/ani16213393/s1: Table S1: The calibration curves for the analysis of RNA modification.

## Abbreviations

The following abbreviations are used in this manuscript:

METTL3	methyltransferase-like 3
METTL14	methyltransferase-like 14
WTAP	wilms tumor 1-associated protein
FTO	fat mass and obesity-associated protein
ALKBH5	alkB homologue 5
YTHDF1	YTH domain family protein 1
YTHDF2	YTH domain family protein 2
YTHDF3	YTH domain family protein 3
YTHDC1	YTH domain containing protein 1
YTHDC2	YTH domain containing protein 2
m^6^A	N^6^-methyladenosine
