# Supplementary material for: RNA Modification Profiles and m6A Regulatory Patterns in the Lungs of Sheep at Different Altitudes
Source: Animals (Basel). 2026 Oct 27;16(21):3393. doi: 10.3390/ani16213393 (PMC13761495; doi:10.3390/ani16213393)
Supplement: Supplementary file 1 [file animals-16-03393-s001.zip › animals-4599169-supplementary.pdf]

Table S1: The calibration curves for the analysis of RNA modification

Index	Full name	Class	RT	Equation	r	Weighting	LLOQ	ULOQ
rC	cytidine	canonical nucleosides	1.12	y = 9.22666e4 x + 7.87406e4	0.99532	1 / x	1	1000
rU	uridine	canonical nucleosides	1.50	y = 3.36535e5 x + 3.18127e5	0.99468	1 / x	1	1000
rG	guanosine	canonical nucleosides	2.42	y = 3.34525e4 x + 26371.20943	0.99560	1 / x	1	1000
rA	adenosine	canonical nucleosides	3.40	y = 1.17202e6 x + 7.99017e5	0.99636	1 / x	1	1000
m3C	3-methylcytidine	modifications	1.12	y = 1.84500e7 x + 9077.41244	0.99918	1 / x	0.001	2
hm5C	5-hydroxymethylcytidine	modifications	1.12	y = 2.70967e7 x + 11698.61734	0.99677	1 / x	0.001	2
Y	pseudouridine	modifications	1.13	y = 3.99440e6 x + 10188.26439	0.99838	1 / x	0.05	2
m1A	1-methyladenosine	modifications	1.16	y = 1.68583e8 x + 9342.41407	0.99990	1 / x	0.001	2
ca5C	5-carboxycytidine	modifications	1.23	y = 8.05262e6 x + 3713.39491	0.99759	1 / x	0.005	2
ho5U	5-hydroxyuridine	modifications	1.27	y = 4.20297e5 x + 1232.83165	0.99919	1 / x	0.05	2
m5C	5-methylcytidine	modifications	1.30	y = 2.74035e7 x + 7814.31975	0.99910	1 / x	0.001	2
m7G	7-methylguanosine	modifications	1.44	y = 5.19879e7 x - 8666.89679	0.99990	1 / x	0.001	2
hm5U	5-hydroxymethyluridine	modifications	1.50	y = 2.16810e5 x + 16776.05361	0.99706	1 / x	0.05	2
m1Y	1-methylpseudouridine	modifications	1.52	y = 1.28619e6 x + 3079.38255	0.99875	1 / x	0.05	2
s2C	2-thiocytidine	modifications	1.64	y = 5.05869e6 x - 6.13265e5	0.99565	1 / x	0.01	2
Cm	2'-O-methylcytidine	modifications	1.71	y = 1.77404e7 x + 12578.84085	0.99865	1 / x	0.001	2
m6U	6-methyluridine	modifications	2.00	y = 2.90465e5 x - 2004.36390	0.99811	1 / x	0.05	2
I	inosine	modifications	2.27	y = 2.31462e6 x + 23678.09920	0.99573	1 / x	0.005	2
m4Cm	N4,2'-O-dimethylcytidine	modifications	2.43	y = 3.89533e7 x + 8465.76990	0.99887	1 / x	0.001	2
m5Cm	5,2'-O-dimethylcytidine	modifications	2.67	y = 2.50743e7 x + 10494.51808	0.99918	1 / x	0.001	2
m5U	5-methyluridine	modifications	2.67	y = 2.57650e6 x + 18008.05653	0.99762	1 / x	0.01	2
mo5U	5-methoxyuridine	modifications	2.74	y = 2.45385e6 x + 15348.29987	0.99813	1 / x	0.01	2
f5C	5-formylcytidine	modifications	2.77	y = 2.93449e6 x + 12478.00914	0.99716	1 / x	0.005	2
s2U	2-thiouridine	modifications	3.07	y = 1.25571e6 x - 26504.56027	0.99838	1 / x	0.05	2
X	xanthosine	modifications	3.20	y = 1.62631e6 x + 254.42700	0.99856	1 / x	0.005	2
Um	2'-O-methyluridine	modifications	3.20	y = 7.46160e5 x + 10411.76361	0.99479	1 / x	0.05	2
m7s6G	7-methyl-6-thioguanosine	modifications	3.27	y = 1.68230e6 x - 2.15191e5	0.99303	1 / x	0.05	2
m3U	3-methyluridine	modifications	3.47	y = 3.25579e6 x + 11533.15108	0.99795	1 / x	0.01	2
s4U	4-thiouridine	modifications	3.54	y = 3.15127e5 x - 21567.07070	0.99878	1 / x	0.01	2
m1I	1-methylinosine	modifications	3.76	y = 1.62250e6 x + 7450.19907	0.99781	1 / x	0.005	2
Im	2'-O-methylinosine	modifications	3.77	y = 3.06715e6 x + 10731.16909	0.99877	1 / x	0.01	2
m1G	1-methylguanosine	modifications	3.80	y = 1.33499e8 x + 3.37974e4	0.99787	1 / x	0.002	2
Gm	2'-O-methylguanosine	modifications	3.85	y = 1.59949e7 x + 4990.73218	0.99621	1 / x	0.002	2
I5C	5-iodocytidine	modifications	3.88	y = 1.46987e7 x + 4011.89039	0.99745	1 / x	0.002	2
mcm5U	5-methoxycarbonylmethyluridine	modifications	4.07	y = 1.03218e7 x + 11983.06398	0.99639	1 / x	0.01	2
m2G	N2-methylguanosine	modifications	4.21	y = 4.03365e7 x + 6228.94131	0.99951	1 / x	0.002	2
ac4C	N4-acetylcytidine	modifications	4.32	y = 4.48779e6 x + 1638.85737	0.99610	1 / x	0.005	2
m2_2_7G	N2,N2,7-trimethylguanosine	modifications	4.35	y = 1.81892e8 x + 27123.19804	0.99780	1 / x	0.001	2
8-oxoA	8-hydroxyadenosine	modifications	4.56	y = 3.41511e7 x + 12087.82449	0.99821	1 / x	0.005	2
5-dA	5'-deoxyadenosine	modifications	4.94	y = 2.96542e6 x + 5218.32117	0.99810	1 / x	0.005	2
Am	2'-O-methyladenosine	modifications	5.04	y = 1.07358e8 x + 5860.90284	0.99943	1 / x	0.001	2
m5s2U	5-methyl-2-thiouridine	modifications	5.05	y = 2.53081e6 x - 11073.92381	0.99191	1 / x	0.02	2
ac6A	N6-acetyladenosine	modifications	5.11	y = 2.41047e7 x + 5820.85025	0.99908	1 / x	0.001	2
m5Um	5,2'-O-dimethyluridine	modifications	5.19	y = 2.38745e6 x + 3487.80449	0.99666	1 / x	0.01	2
f5Cm	5-formyl-2'-O-methylcytidine	modifications	5.58	y = 5.10391e6 x - 760.45349	0.99701	1 / x	0.005	2
m2_2G	N2,N2-dimethylguanosine	modifications	5.83	y = 5.75268e7 x + 9079.30194	0.99857	1 / x	0.001	2
m6A	N6-methyladenosine	modifications	6.20	y = 2.46352e8 x + 5.35880e4	0.99825	1 / x	0.001	2
ac4Cm	N4-acetyl-2'-O-methylcytidine	modifications	6.46	y = 7.26464e6 x + 1459.00682	0.99736	1 / x	0.005	2
mcm5s2U	5-methoxycarbonylmethyl-2-thiouridine	modifications	6.50	y = 7.84922e6 x - 2.69806e5	0.99372	1 / x	0.005	2
m6G	O6-methylguanosine	modifications	6.57	y = 8.14296e7 x + 7578.71003	0.99940	1 / x	0.001	2
s2Um	2-thio-2'-O-methyluridine	modifications	6.73	y = 7.29739e5 x - 11954.85639	0.99757	1 / x	0.01	2
m6Am	N6,2'-O-dimethyladenosine	modifications	6.85	y = 2.25966e8 x + 14100.45812	0.99486	1 / x^2	0.001	2
t6A	N6-threonylcarbamoyladenosine	modifications	6.90	y = 2.60961e7 x + 15374.51599	0.99915	1 / x	0.001	2
5-dmsA	5'-Deoxy-5'-methylthioadensine	modifications	7.00	y = 4.81793e7 x - 3.81091e4	0.99889	1 / x	0.001	2
m6_6A	N6,N6-dimethyladenosine	modifications	7.17	y = 2.90711e8 x + 19504.39804	0.99857	1 / x^2	0.0005	2
cis-io6A	N6-(cis-hydroxyisopentenyl)adenosine	modifications	7.29	y = 1.95084e8 x + 5.62022e4	0.99683	1 / x	0.0005	2
trans-io6A	trans-ribosylzeatin	modifications	7.41	y = 1.89457e8 x + 3.37379e4	0.99879	1 / x	0.0005	2
m6_6Am	N6,N6,2'-O-trimethyladenosine	modifications	7.66	y = 2.81137e8 x + 27541.64174	0.99308	1 / x^2	0.0005	2
i6A	N6-isopentenyladenosine	modifications	8.62	y = 1.27608e8 x + 6137.54235	0.99453	1 / x^2	0.00005	2
